# Influence of food matrix type on extracellular products of *Vibrio parahaemolyticus*

**DOI:** 10.1186/s12866-018-1207-7

**Published:** 2018-07-05

**Authors:** Rundong Wang, Lijun Sun, Yaling Wang, Yijia Deng, Zhijia Fang, Ying Liu, Qi Deng, Dongfang Sun, Ravi Gooneratne

**Affiliations:** 1 College of Food Science and Technology, Guangdong Provincial Key Laboratory of Aquatic Product Processing and Safety, Key Laboratory of Advanced Processing of Aquatic Products of Guangdong Higher Education Institution, Guangdong Ocean University, Zhanjiang, 524088 China; 2 Centre for Food Research and Innovation, Department of Wine, Food and Molecular Biosciences, Lincoln University, Lincoln, Canterbury, 7647 New Zealand

**Keywords:** *Vibrio parahaemolyticus*, Food matrices, Extracellular products, Pathogenicity

## Abstract

**Background:**

Two strains of *Vibrio parahaemolyticus* (ATCC 17802 and 33847) in shrimp, oyster, freshwater fish, pork, chicken and egg fried rice were evaluated for production of hemolysin and exoenzymes of potential importance to the pathogenicity of this bacterium.

**Results:**

The two strains of *V. parahaemolyticus* produced hemolysin, gelatinase, caseinase, phospholipase, urease, DNase and amylase in selected food matrices. Significantly higher (*p <* 0.05) hemolytic activity was produced by *V. parahaemolyticus* in egg fried rice > shrimp > freshwater fish > chicken > oyster > pork. But the exoenzyme activities were not consistent with the hemolytic activity profile, being significantly higher (*p <* 0.05) in shrimp > freshwater fish > chicken > oyster > pork > egg fried rice. Filtrates of *V. parahaemolyticus* from shrimp, freshwater fish and chicken given intraperitoneally to adult mice induced marked liver and kidney damage and were highly lethal compared with the filtrates of *V. parahaemolyticus* from oyster > egg fried rice > pork.

**Conclusion:**

From in vitro and in vivo tests, it appears that the food matrix type has a significant impact on the activity of extracellular products and the pathogenicity of *V. parahaemolyticus*. From a food safety aspect, it is important to determine which food matrices can stimulate *V. parahaemolyticus* to produce additional extracellular factors. This is the first report of non-seafood including freshwater fish and chicken contaminated with *V. parahaemolyticus* to have been shown to be toxic to mice in vivo.

## Background

*Vibrio parahaemolyticus* is a gram-negative, facultative, anaerobic, halophilic bacterium that inhabits marine or estuarine environments [1, 2]. The natural host for this bacterium is variable because it lives in water and is concentrated in shellfish which can serve as reservoirs [3–5]. *V. parahaemolyticus* can contaminate raw or undercooked shrimp, fish, oyster and cause abdominal pain, acute gastroenteritis, diarrhea, and infection by the O3: K6 pandemic strain resulted in a massive number of human deaths [6–8] in several countries including China, Japan and the United States [9–11].

Following contamination of food with *V. parahaemolyticus*, both the bacterial cells and extracellular products contribute to the pathogenicity and among them, the extracellular products play a dominant role [12–14]. Of all extracellular products, hemolysin (thermostable direct hemolysin, thermostable-related hemolysin) is regarded as the most important virulence factor, and controls a variety of biological activities including hemolytic activity, cytotoxicity, and enterotoxicity [15–17], besides other factors such as exoenzymes [18, 19]. Among these, gelatinase and caseinase belong to a family of proteolytic enzymes that can cause tissue damage and hydrolyze various protein substrates including hemoglobin and other small amounts of biologically active peptides [20, 21]. Phospholipases involved in nutrient acquisition through the degradation of membrane lipids may also cause harm to the host [22]. DNase can act as endonuclease and contribute to DNA hydrolysis, amylase can hydrolyze carbohydrate to provide energy for the growth of *V. parahaemolyticus* [23] and urease may act as hemolysin [24].

Seafood has long been considered to be the only carrier of *V. parahaemolyticus*. Therefore, from a food safety aspect, more attention has been paid to seafood products. However, there is new evidence that *V. parahaemolyticus* can also contaminate non-seafood matrices (a prevalence of ~ 32.5%) such as poultry, pork, freshwater fish, eggs and their products including egg fried rice, by cross contamination of seafood to non-seafood and via cooking utensils [25–27], which suggest that *V. parahaemolyticus* can also cause food infection via many non-seafood types. Our previous studies [28] found that the virulence factors of *V. parahaemolyticus* can trigger high or low pathogenicity in different foods. But, little is known of the composition of extracellular products in different food matrices.

To better assess the risk of *V. parahaemolyticus* in different food matrices, a clearunderstanding of the extracellular products is essential. In this study, we examined the importance of extracellular products, hemolysins, gelatinase, caseinase, phospholipase, urease, DNase and amylase to the pathogenicity of *V. parahaemolyticus* in selected seafood and non-seafood products and tested their combined pathogenicity in a mouse model.

## Methods

### Bacterial strains and growth conditions

*V. parahaemolyticus* strains ATCC 17802 and ATCC 33847 were stored in 25% glycerol at − 20 °C. Each strain was grown in brain heart infusion (BHI) broth (BLBT, Beijing, China) containing 3% NaCl, at 37 °C for 24 h. The inoculum was thrice passaged in BHI-3% NaCl. The final concentration of inoculants were adjusted to ~ 10^4^ CFU/ml and used to inoculate the food matrices.

### Food matrices preparation and inoculation

Shrimp (*Litopenaeus vannamei*), oyster (*Crassostrea*), freshwater fish (*Tilapia*), pork and chicken were purchased from a local supermarket in Zhanjiang, China, and the meat was used in the study. Egg fried rice (rice:egg = 1:1) was cooked at 80 °C for 20 min, in the laboratory.

Test portions, 100 g each (*n* = 3) of shrimp meat, oyster, freshwater fish meat, pork, chicken, and egg fried rice, added salt at 3% in sterile Erlenmeyer flasks were sterilized by autoclave (YXQ-L-50A, Shanghai Boxun, Shanghai, China) at 100 °C for 20 min to kill native bacteria. Then each sample was inoculated with either 1 mL of the final *V. parahaemolyticus* ATCC 17802 or ATCC 33847 (described above, cell number ~ 10^3^ CFU/g). Inoculated samples were mixed thoroughly in a vortex mixer (XW-80A, Qilinbei, Haimen, China) for 10 min and incubated at 37 °C until the bacterial counts were approximately 10^9^ CFU/g.

After incubation, the inoculated food samples were separately washed with 100 mL 0.01 M phosphate-buffered saline (PBS, pH 7.2), and the solution centrifuged (Thermo Lynx 6000, Thermo Scientific, Waltham, MA) at 12000 rpm for 20 min at 4 °C. The supernatants were filtered (0.22 μm, Millipore, Billerica, MA) and stored at − 20 °C until use. The control food matrix samples were subjected to the same procedure except that these samples were not inoculated with *V. parahaemolyticus*.

### Hemolytic activity

The relative hemolytic activity test measured the total hemolysins in the samples and were detected as described by Takamatsu et al. [29] modified by Jiang et al. [30]. Rabbit hemocytes were obtained by centrifugation of blood (3500 rpm, 4 °C, 5 min) three times (washed with PBS each time) and diluted to 5% with PBS. Subsequently, a sample (400 μL) of each food matrix was mixed with 100 μL of 5% rabbit red blood cells in 1.5-mL sterile tubes and incubated at 37 °C for 1.5 h. Unlysed erythrocytes were allowed to pelletize overnight at 4 °C, then 200-μL portions of the supernatant were transferred to 96-well flat-bottomed microplates (Nunc, Thermo Scientific, Waltham, MA) and the absorbance measured at 570 nm with a microplate reader (Varioskan Flash, Thermo Scientific, Waltham, MA). For controls, the same procedure was employed except the samples were changed to food matrix filtrates without the *V. parahaemolyticus* inocula*.* The results are reported as: A_relative hemolytic activity_ = A_sample_ − A_control_.

### Production of extracellular enzymes

In separate plates, 0.5% (*w*/*v*) gelatin [31], 0.2% (w/v) casein [32], 3% (*v*/v) egg yolk emulsion, 2.5% (w/v) urea [31], 0.01% (w/v) toluidine blue or 0.2% (w/v) soluble starch [14] were added to tryptone soya agar (TSA) to determine gelatinase, caseinase, phospholipase, urease, DNase and amylase enzyme activities.

The exoenzyme activities of sample and control filtrates were determined by the Oxford Cup Method [33]. Briefly, 180 μL of the filtrate in triplicate were added into the Oxford cups in TSA plates containing different substrates. All the plates were incubated at 37 °C for 12 h. The positive reaction of a clear halo was detected with gelatinase and caseinase following addition of 70% trichloroacetic acid. The positive reaction to phospholipase, urease, and DNase were characterized by the presence of opaque halo, yellow halo, and pink halo respectively. To detect amylase, 5 mmol/L KI-I_2_ solution was added to the TSA plates after the 12-h incubation and a clear halo indicated a positive reaction. All positive zones around the cup were measured.

### Mice pathogenicity test

#### Lethality study

One hundred and eight female KM mice (20 ± 2 g, 6 weeks old) were obtained from Animal Center of Guang Dong Province. During the experimental period, mice were reared under standard laboratory conditions (12 h light-dark cycle, temperature of 20 ± 1 °C, humidity 60 ± 5%) in 18 stainless steel cages with free access to distilled water and sterilized food. The mice were acclimatized to this environment for 5 days randomly assigned to 18 groups (*n* = 6). Twelve experimental groups were injected with millipore-filtered food matrix filtrates contaminated with either *V. parahaemolyticus* ATCC 17802 or ATCC 33847 strain, and six control groups with control food matrix filtrates, intraperitoneally (i.p.) at 0.2 mL / 10 g body weight (bw). The mortality rate of mice was recorded for 48 h.

#### Biochemical indices

For biochemical studies, another 108, 6-week-old female KM mice (obtained from Animal Center of Guang Dong Province) were injected i.p. with *V. parahaemolyticus* food matrix filtrates as per the above described protocol. Mice were euthanized by exsanguination while under ether vapor narcosis (in a funnel) at 12 h. Blood was sampled by percutaneous cardiac puncture and centrifuged at 3500 rpm for 10 min to obtain serum for detection of three liver-specific enzymes (aspartate aminotransferase (AST), alanine aminotransferase (ALT) and alkaline phosphatase (ALP)) and the kidney-specific enzyme blood urea nitrogen (BUN), using detection kits (Nanjing Jiancheng Bioengineering Institute, Nanjing, China), to assess tissue damage.

#### Ethics approval and consent to participate

All mouse experiments were conducted according to the guidelines provided by the.

Animal Care and Welfare Committee of Guangdong Ocean University (License Number: SYXK 2014–0053).

### Statistical analysis

All data were analyzed using the software SPSS 19.0 (SPSS Inc., Chicago, IL, USA). Differences between the means were tested by one-way ANOVA, with the level of significance set at *p <* 0.05.

## Results

### Hemolytic activity

**Fig. 1:**
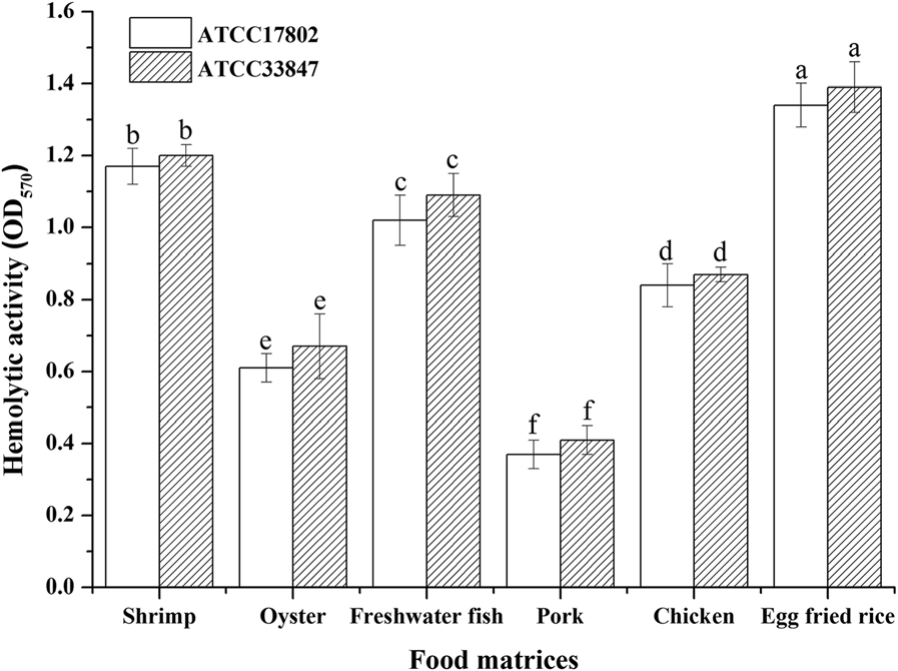
Hemolytic activity of *V. parahaemolyticus* in different food matrices. Error bars represent standard deviations of mean values from triplicate experiments (Control groups excluded). Means with different lowercase letters were significantly different (*p <* 0.05) among different food matrices

The relative absorbance of different food matrices filtrates reflected the hemolytic activity of *V. parahaemolyticus* in food samples. The *V. parahaemolyticus* ATCC 33847 showed a higher hemolytic activity than ATCC 17802 in all selected food samples. Irrespective of the *V. parahaemolyticus* strain, the hemolytic activity was significantly higher in egg fried rice > shrimp > freshwater fish > chicken > oyster > pork (Fig. 1).

### Production of extracellular enzymes

ATCC 17802 and ATCC 33847 strains were tested for six exoenzymes previously reported to be responsible for *V. parahaemolyticus* virulence. The two pathogenic strains of *V. parahaemolyticus* produced a wide variety of extracellular enzymes including gelatinase, caseinase, phospholipase, urease, DNase and amylase in the selected food matrices (Table 1). Extracellular factor activity was generally higher with the ATCC 33847 strain than with ATCC 17802. Overall, both *V. parahaemolyticus* strains produced significantly high activities (*p* < 0.05) of gelatinase, caseinase, phospholipase, urease, DNase and amylase in shrimp > freshwater fish > chicken > oyster > pork > egg fried rice.

**Table 1:** Extracellular enzyme composition and activity of *Vibrio parahaemolyticus* in different food matrices^f^

Measurement index	Enzyme	Strains	Food matrix					
			Shrimp	Oyster	Freshwater fish	Pork	Chicken	Egg fried rice
Positive circle diameter (mm)	Gelatinase	ATCC 17802	21.98 ± 2.10^a^	15.92 ± 1.39^d^	20.72 ± 1.98^b^	15.00 ± 1.67^e^	17.52 ± 1.44^c^	16.56 ± 1.12^c^
		ATCC 33847	29.42 ± 1.71^a^	17.20 ± 1.39^c^	21.12 ± 1.45^b^	14.88 ± 1.56^e^	18.29 ± 0.99^c^	16.16 ± 0.91^d^
	Caseinase	ATCC 17802	25.62 ± 2.25^a^	18.16 ± 1.41^c^	20.32 ± 1.32^b^	17.10 ± 1.08^d^	24.40 ± 3.06^a^	13.94 ± 2.64^e^
		ATCC 33847	26.20 ± 1.19^a^	18.08 ± 0.99^c^	22.80 ± 0.92^b^	12.60 ± 1.52^e^	19.40 ± 1.38^c^	14.40 ± 2.58^d^
	Phospholipase	ATCC 17802	14.90 ± 1.24^b^	9.20 ± 1.31^e^	12.50 ± 0.79^c^	14.04 ± 1.10^b^	14.12 ± 0.27^b^	16.80 ± 1.04^a^
		ATCC 33847	16.78 ± 0.49^b^	10.00 ± 1.39^d^	17.00 ± 0.52^b^	13.80 ± 1.53^c^	13.30 ± 1.05^c^	18.20 ± 0.89^a^
	Urease	ATCC 17802	21.22 ± 1.23^a^	16.02 ± 1.98^c^	18.46 ± 0.58^b^	15.33 ± 0.74^d^	16.92 ± 0.32^c^	11.58 ± 1.02^e^
		ATCC 33847	24.30 ± 0.69^a^	15.88 ± 1.34^c^	19.60 ± 1.08^b^	15.10 ± 1.19^d^	17.10 ± 0.52^c^	10.94 ± 1.25^e^
	DNase	ATCC 17802	26.28 ± 1.76^a^	12.30 ± 0.96^d^	19.76 ± 1.03^b^	13.54 ± 2.10^d^	16.40 ± 0.99^c^	12.42 ± 1.45^d^
		ATCC 33847	27.90 ± 0.81^a^	13.90 ± 0.68^d^	18.82 ± 1.20^b^	12.42 ± 1.16^e^	17.04 ± 0.36^c^	14.00 ± 1.05^d^
	Amylase	ATCC 17802	18.34 ± 1.11^a^	16.64 ± 1.21^c^	17.08 ± 0.29^b^	15.40 ± 1.08^c^	17.52 ± 0.40^b^	15.30 ± 1.64^c^
		ATCC 33847	20.80 ± 1.62^a^	17.12 ± 0.53^d^	17.99 ± 0.65^c^	13.70 ± 1.58^e^	18.98 ± 0.25^b^	18.20 ± 0.78^c^

Note: ^f^ Mean ± standard deviation of three replicates. Means in the same line with different superscript letters are significantly different (*p* < 0.05). Results were negative for the filtrates of all food matrices not inoculated with *V. parahaemolyticus*

### Mice pathogenicity tests

#### Lethality

The mortality of mice injected with the food matrix filtrates was higher with the shrimp matrix than other food matrices (Table 2) probably because of the higher extracellular enzyme activity). The mortality rate was highest in shrimp > freshwater fish > chicken > oyster > egg fried rice > pork. Strain ATCC 33847 appeared more virulent in that it caused more deaths. There were no deaths in the control groups.

**Table 2:** Mortality in mice injected intraperitoneally with different food matrix filtrates (*n* = 6)

Measurement index	Strains	Food matrix filtrates						
		Shrimp	Oyster	Freshwater fish	Pork	Chicken	Egg-fried rice	Control^a^
Death rate	ATCC17802	2/6	1/6	2/6	0/6	1/6	0/6	0/36
	ATCC33847	3/6	1/6	2/6	0/6	2/6	1/6	

Note: Each mouse was injected intraperitoneally with 0.2 mL / 10 g bw of food matrix filtrate and the death rate recorded at 48 h. ^a^ Control mice were injected with filtrates of shrimp, oyster, freshwater fish, pork, chicken and egg fried rice that were not inoculated with *V. parahaemolyticus*

#### Liver and kidney damage in mice

**Fig. 2:**
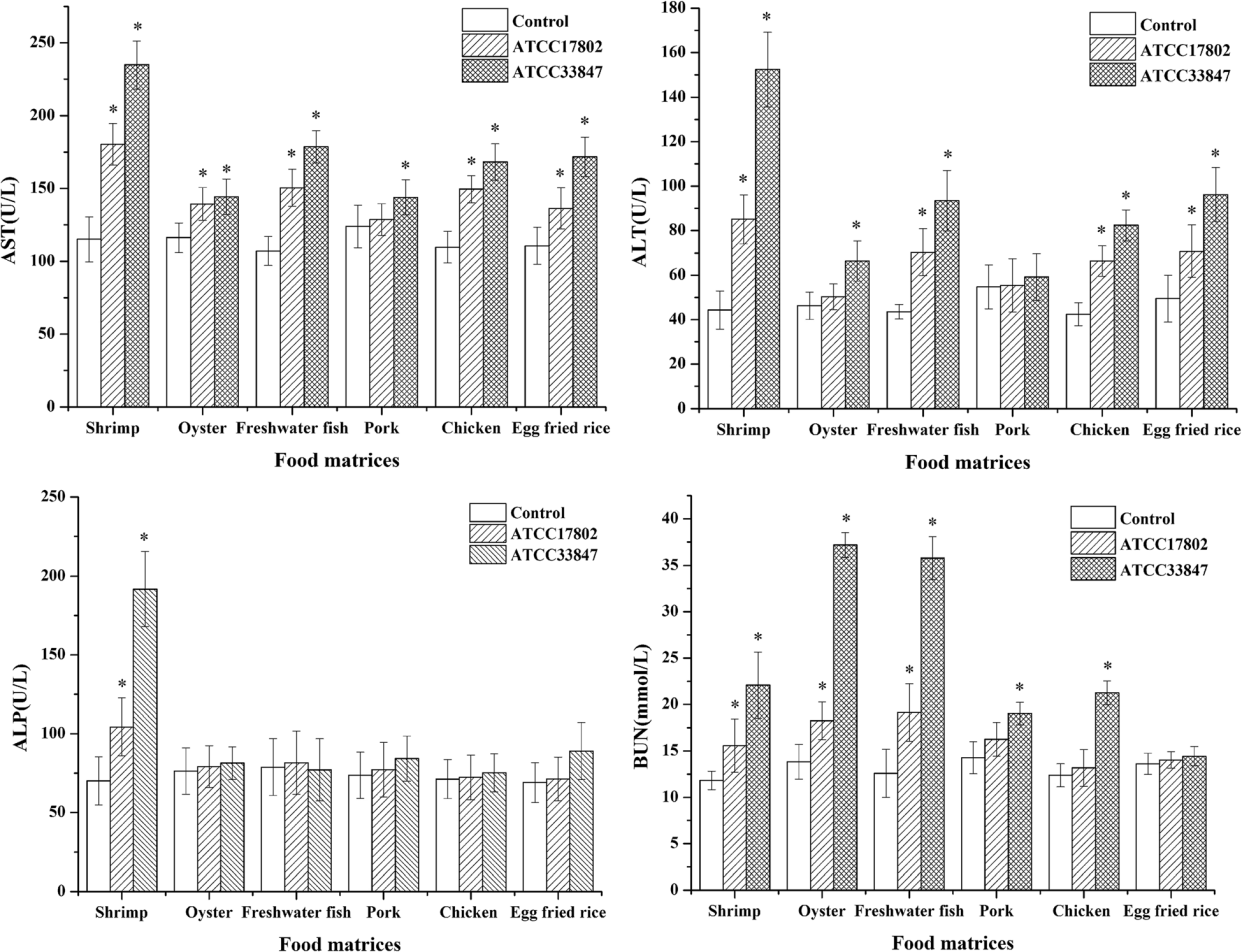
Liver and kidney function indices detection. The mice were sacrificed after giving different food matrix filtrates intraperitoneally for 12 h. The test groups given filtrates of *V. parahaemolyticus* ATCC 17802 (or ATCC33847) samples, control groups given filtrates of foods that were not inoculated with *V. parahaemolyticus*. Means with asterisks (*) are significantly different (*p* < 0.05) from the respective controls.

The serum biochemical parameters indicative of liver and kidney function measured at 12 h in mice injected with different food matrix filtrates are shown in Fig. 2. In pork samples, no significant changes (*p* > 0.05) were detected in most of the parameters compared with the controls. AST and ALT activity indicative of liver damage were significantly higher (*p* < 0.05) in mice given shrimp, freshwater fish, chicken and egg fried rice filtrates compared with the respective controls. ALP was significantly higher (*p* < 0.05) only in mice given shrimp. BUN activity indicative of kidney damage was significantly elevated (*p* < 0.05) in all test mice injected with oyster > freshwater fish > shrimp > chicken > pork > egg fried rice, compared with the respective controls. The food matrix filtrates of ATCC 33847 affected liver and kidney function more than ATCC 17802.

## Discussion

A correlation between virulence and the production of extracellular products by food contaminant bacteria [34–36] including by *V. alginolyticus* [37] and *V. vulnificus* [38] but little is known about the specific extracellular products of *V. parahaemolyticus* and its pathogenicity in different food matrices. To our knowledge, this study is the first to examine the extracellular products – hemolysin and six exo-enzymatic activities in two pathogenic *V. parahaemolyticus* strains in selected seafood and non-seafood and assess relative risk.

Hemolysin is an important virulence factor responsible for the pathogenicity of *V. parahaemolyticus* because it can lyse cells, especially red blood cells, and cause systemic infections [39]. In the hemolytic activity test, the two pathogenic *V. parahaemolyticus* strains produced hemolysin not only in seafood but also in non-seafood. The significantly higher (*p* < 0.05) hemolytic activity observed in egg fried rice than in shrimp >freshwater fish > chicken> oyster > pork (Fig. 1). We hypothesized that the nutrition factors in egg fried rice can also promote *V. parahaemolyticus* to produce more hemolysin, which is in agreement with Taniguchi et al. [40] and Shinoda et al. [41] who identified a lecithin-dependent hemolysin that can also cause hemolysis. So, we believe that the high lecithin concentration in eggs may induce *V. parahaemolyticus* to produce more hemolysin in egg fried rice. This is the first evidence of *V. parahaemolyticus* producing more hemolysin in lecithin-enriched food, which means that some non-seafood may in fact be equally pathogenic than the traditionally affected seafood and therefore worthy of more attention. *V. parahaemolyticus,* like many other bacteria, require a source of iron and its hemolytic activity and virulence are greatly enhanced on exposure to elevated iron concentrations [42, 43]. Hence, we believe that it is also important to pay more attention to monitoring of foods with a higher iron content. Although the mortality rates of mice injected with different food matrix filtrates (containing *V. parahaemolyticus* extracellular products) were highest in shrimp > freshwater fish > chicken > oyster > egg fried rice > pork, it was not possible to prove this statistically because of the limited number of mice used in the study.

*Vibrio* strains are known to produce a series of exoenzymes that contribute to expression of pathogenicity. In this study, no differences were observed in the composition of exoenzymes between the two pathogenic *V. parahaemolyticus* strains in different food matrices, which means that there is a high food safety risk no matter what food matrix type is contaminated by *V. parahaemolyticus* [44].

Results from our study suggest that *V. parahaemolyticus* produce significantly higher activity (*p* < 0.05) of gelatinase, caseinase, urease, DNase and amylaes in shrimp matrix than freshwater fish (Table 2) and are in agreement with the results of Liu et al. [31] and Zhang and Austin [32] who reported that higher phospholipase, gelatinase and caseinase activities were detected in *Vibrio* species isolated from marine shrimp, fish, and shark. The virulence of pathogenic *Vibrios* is related to their ability to produce exoenzymes [45]*.* As shown in Table 1, the exoenzyme activities in chicken were greater than in the oyster matrix, which suggested that *V. parahaemolyticus* produced more exoenzymes in the chicken and hence that some non-seafoods also pose a high risk to humans. The lower exoenzyme activities observed in pork and egg fried rice (Table 1) are interesting because Iuchi and Tanaka [46] showed that production of exoenzymes in *V. parahaemolyticus* was repressed by various carbohydrates present in the medium. We believe that the high concentration of carbohydrates in egg fried rice may have suppressed *V. parahaemolyticus*’s ability to secrete exoenzymes. Analyzing the activities of different exoenzymes in different food matrices provides a way to comprehensively study the pathogenic mechanism of *V. parahaemolyticus.* However, further studies are required to determine which factor(s) have the most influence on the production of exoenzymes in pork and chicken.

Although cytotoxicity assays [15, 47] are often used to study the pathogenicity of *vibrio* extracellular products, in vitro tests do not adequately represent the true toxicity in vivo [48]. In our study, the mouse model was used to determine the toxicity of *V. parahaemolyticus* extracellular products. It was observed that shrimp filtrate was highly lethal to adult mice (Table 2) and caused more damage to liver and kidney (Fig. 2) than other food matrix filtrates, followed by freshwater fish and chicken filtrates. It was interesting to observe that egg fried rice, which showed the highest hemolytic activity, did not cause significant pathogenicity to mice. This is in contrast to the traditional view that hemolysin is the major virulence factors of *V. parahaemolyticus* and that the high hemolytic activity is responsible for most of the tissue damage [49]. We believe that the pathogenicity of *V. parahaemolyticus* extracellular products is dependent not only on hemolysin, but also on the mixture of other secreted enzymes. Xu et al. [13] and Bhattacharjee et al. [50] demonstrated that pathogenic *V. parahaemolyticus,* although lacking hemolysin, can still cause cytotoxicity and death in mice. Other studies have also suggested that hemolysin is not necessarily the only virulence factor of pathogenic *V. parahaemolyticus* [39, 51]. Liver and kidney damage, as shown by elevated clinical chemistry indices such as ALT, ALP, AST and BUN activity (Fig. 2), were observed in mice given shrimp filtrate and to a lesser extent in mice given pork or egg-fried-rice filtrates. According to Maeda and Yamamoto [52], the high levels of exoenzyme activity alone could cause extensive damage to host tissue. In addition, damage to spleen and stomach were observed in mice given shrimp filtrate (unpublished observation). Our mouse results are in agreement with the findings of Moreno and Landgraf [38] and provide further proof that exoenzymes play a vital role in the pathogenicity of *V. parahaemolyticus.* Hence it is important to consider the extracellular enzymes activities also in risk assessment. Besides, the type III secretion (T3SS) system of *V. parahaemolyticus* also play a role in lethality in the murine infection model [14] although the mechanism of action of the T3SS system that influences the virulence is not well understood.

Baffone and others [34, 53] demonstrated that most of the extracellular products identified in *V. alginolyticus* and *V. vulnificus* are not directly associated with pathogenicity but require the bacterial cells also to be present to cause pathogenicity, unlike *V. parahaemolyticus* where the extracellular products alone can be pathogenic. In our studies also, the extracellular products of *V. parahaemolyticus* alone were pathogenic to mice. It is suggested that the pathogenesis mechanism of *V. parahaemolyticus* is different to other *Vibrio* types*.* Besides the invasion damage caused by the bacteria, the virulence factors of *V. parahaemolyticus* are highly toxic to tissues. If the food matrices are contaminated by *V. parahaemolyticus*, transient heating could remove most of the bacteria, but some thermo-tolerant products including thermostable direct hemolysin, thermostable related hemolysin, and other thermo-tolerant enzymes that can survive at 85 °C for 10 min [54] would possess biological activity to induce tissue damage. Hence, food should be heated to at least 85 °C for 10 min to destroy the activity of pathogenic thermo-tolerant products of *V. parahaemolyticus*. Besides, the food producers could incorporate probiotics (eg: *Lactobacillus pentosus*, *Streptomyces*) [55, 56] to inhibit the growth of *V. parahaemolyticus* and reduce the production of pathogenic extracellular products. If humans are infected with *V. parahaemolyticus*, bacteriophage therapy [57, 58] could be used to control and inhibit the virulence of *Vibrio* species. Such methods can be regarded as better strategies in view of the ever-increasing anti-microbial resistance in both humans and animals.

## Conclusions

The present study suggests that the food matrix type has a marked effect on the pathogenicity of extracellular products of *V. parahaemolyticus.* Higher hemolytic activity observed in egg fried rice is an important new finding from a food safety aspect. Significantly higher activity of exoenzymes observed in shrimp and freshwater fish was strongly linked to high pathogenicity. This is the first report to show that besides the extracellular products in shrimp produced by *V. parahaemolyticus*, some non-seafood such as chicken infected with *V. parahaemolyticus* may also be toxic to mice in vivo. Although, for non-seafood matrices such as chicken it is unlikely that high levels of *V. parahaemolyticus* could be reached by cross-contamination from seafood matrices or via cooking utensils, the high pathogenicity still exists and need to be paid attention. It appears that exoenzymes, in addition to hemolysin, are involved in the pathogenesis of *V. parahaemolyticus* in food matrices.
